# Oral and IV Varespladib Rescue Experiments in Juvenile Pigs with Weakness Induced by Australian and Papuan *Oxyuranus scutellatus* Venoms

**DOI:** 10.3390/toxins15090557

**Published:** 2023-09-07

**Authors:** Lyndi L. Gilliam, John Gilliam, Stephen P. Samuel, Rebecca W. Carter, Jerry Ritchey, Tommaso Bulfone, José María Gutiérrez, David J. Williams, Daniela M. Durkin, Sally I. Stephens, Matthew R. Lewin

**Affiliations:** 1 Department of Veterinary Clinical Sciences, Center for Veterinary Health Sciences, Oklahoma State University, Stillwater, OK 74078, USA; l.gilliam@okstate.edu (L.L.G.); john.gilliam@okstate.edu (J.G.); jerry.ritchey@okstate.edu (J.R.); 2 Division of Research Ophirex, Inc., Corte Madera, CA 94925, USA; stephen@ophirex.com (S.P.S.); rebecca@ophirex.com (R.W.C.); sally@ophirex.com (S.I.S.); 3 Center for Exploration and Travel Health, California Academy of Sciences, San Francisco, CA 94118, USA; tommaso.bulfone@ucsf.edu (T.B.);; 4 School of Medicine, University of California, San Francisco, CA 94143, USA; 5 Instituto Clodomiro Picado, Facultad de Microbiología, Universidad de Costa Rica, San José 11501-2060, Costa Rica; josemorama@gmail.com; 6 Regulation and Prequalification Department (RPQ) at the World Health Organization (WHO), 1211 Geneva, Switzerland; williamsd@who.int

**Keywords:** snakebite, *Oxyuranus*, coastal taipan, venom, antivenom, phospholipase A2 inhibitors, direct toxin inhibitor, varespladib

## Abstract

Antivenom is currently the standard-of-care treatment for snakebite envenoming, but its efficacy is limited by treatment delays, availability, and in many cases, species specificity. Many of the rapidly lethal effects of envenoming are caused by venom-derived toxins, such as phospholipase A2 (sPLA2); therefore, small molecule direct toxin inhibitors targeting these toxins may have utility as initial and adjunct therapies after envenoming. Varespladib (intravenous, IV) and varespladib-methyl (oral) have been shown to potently inhibit sPLA2s from snake venoms in murine and porcine models, thus supporting their further study as potential treatments for snakebite envenoming. In this pilot study, we tested the ability of these compounds to reverse neurotoxic effects of venom from the Australian and Papuan taipan (*Oxyuranus scutellatus*) subspecies in juvenile pigs (*Sus domesticus*). The mean survival time for control animals receiving Australian taipan venom (0.03 mg/kg, *n* = 3) was 331 min ± 15 min; for those receiving Papuan taipan venom (0.15 mg/kg, *n* = 3) it was 178 ± 31 min. Thirteen pigs received Australian taipan venom and treatment with either IV or oral varespladib (or with IV to oral transition) and all 13 survived the duration of the study (≥96 h). Eight pigs received Papuan taipan venom followed by treatment: Briefly: Two animals received antivenom immediately and survived to the end of the study. Two animals received antivenom treatment delayed 45 min from envenoming and died within 4 h. Two animals received similarly delayed antivenom treatment and were rescued by varespladib. Two animals were treated with varespladib alone after a 45-min delay. Treatment with varespladib only was effective but required repeat dosing over the course of the study. Findings highlight both the importance of early treatment and, as well, a half-life for the investigational inhibitors now in Phase II clinical trials for snakebite. Varespladib rapidly reversed weakness even when administered many hours post-envenoming and, overall, our results suggest that varespladib and varespladib-methyl could be efficacious tools in the treatment of sPLA2-induced weakness from *Oxyuranus* envenoming. Further clinical study as initial therapy and as potential method of rescue from some types of antivenom-resistant envenomings are supported by these data.

## 1. Introduction

At least five million people are affected by snakebite every year. 5.8 billion people live within proximity to venomous snake habitats and are therefore at risk of contributing to the previous statistic [1]. Snakebite is a severe risk for children at play [2] and an occupational hazard for agricultural workers and other occupations or avocations occurring in rural locales that do not have rapid access to emergency medical care. As a result, antivenom administration is frequently infeasible or delayed, becoming ineffective at reversing neurotoxic effects if not given promptly following envenoming [3,4,5,6]. In addition to cost, antivenoms such as CSL taipan-specific antivenom used in this study require refrigeration, limiting its storage options in resource-limited settings [7,8,9].

These persistent challenges describe an unmet need for rapid, broad-spectrum, heat-stable, low-cost, easy-to-administer treatments applicable without snake identification or even certainty about the severity of the bite. If such therapies were available, they might also be used as adjuncts to positively influence outcomes early while enhancing the performance of antivenoms in the hospital setting as well. 

In humans, rapidly lethal neurotoxic and long-term effects seen after envenoming are most often attributed to the effects of venom-derived phospholipase A2 (sPLA2) and other neurotoxins such as three-finger toxins (3-FTx) and dendrotoxins [10,11,12,13,14,15]. Venom components vary across species—neurotoxicity from viper venoms is predominantly sPLA2-based with a complete absence of 3-FTx [16], whereas the venom of many elapids such as cobras, kraits, and sea snakes contain both sPLA2s and 3-FTx. The neurotoxic effects of mambas are caused by dendrotoxins and unique 3-FTx with anticholinesterase activity [11]. In taipan venoms, sPLA2s are thought to cause damage to the presynaptic neuromuscular junction as well as play a key role in other clinical effects such as hemodynamic instability [8,17,18,19,20,21,22,23,24,25,26]. As such, snake venom sPLA2 enzymes and non-enzymatic sPLA2-like proteins are appealing therapeutic targets because of their involvement in many important early and late pathological features of envenoming, including acute neurotoxicity, myotoxicity, anticoagulant effects, hemolysis, and severe alterations in blood pressure [27,28,29]. Candidate molecules varespladib and its prodrug varespladib-methyl (LY315920 and LY333013, respectively) inhibit venom sPLA2s and may have favorable attributes of safety, spectrum of effect, potency, and low cost of goods resulting from its entirely synthetic manufacturing. Both varespladib and varespladib-methyl are currently in Phase II clinical trials for snakebite envenoming [7,30,31]. 

Varespladib and varespladib-methyl have been shown to potently inhibit venom sPLA2s from multiple snake species [32] and have been identified as potential treatment tools for snakebite envenoming [32,33,34,35]. Preclinical efficacy of these drugs has also been demonstrated in the experimental rescue of juvenile pigs and mice from lethal envenoming by the Eastern coral snake (*Micrurus fulvius*) and Papuan taipan (*Oxyuranus scutellatus*), respectively [35,36]. With respect to Papuan taipan venom, previous mouse study results showed that a single dose of varespladib-methyl administered with a 60 min delay following a lethal dose of venom resulted in the survival of seven out of nine mice, whereas only one out of five mice receiving antivenom with a 60 min delay survived [35]. To extend this work on Papuan taipan envenoming [35,37], we tested varespladib and its prodrug varespladib-methyl in a juvenile pig model against lethal envenoming with coastal taipan (*Oxyuranus scutellatus*) venoms from and Papua New Guinea. Australian and Papuan coastal taipan venoms were selected because their main neurotoxicity appears to rely on the action of potent presynaptically-acting neurotoxic, heterotrimeric sPLA2 taipoxins [26,38,39,40,41,42]. These venoms are known to induce neurotoxicity and also hemodynamic instability, myopathy, and procoagulant effects resulting in, for example, pulmonary emboli [41]. The current series of pilot experiments were intended to assess the ability of varespladib with and without antivenom to reverse venom-induced weakness and prolong survival. Various dosing and timing regimens of varespladib administration were tested. 

## 2. Results

### 2.1. Venom Dose Finding

**Figure 1:**
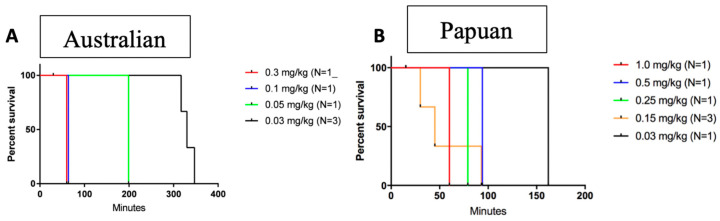
Venom dose finding in preparation for treatment with antivenom and experimental therapeutic regimens with varespladib. An amount of 0.03 mg/kg was selected for Australian taipan venom (**A**) and 0.15 mg/kg for Papuan taipan (**B**). Venom dose selection was fit-for-purpose and was not intended to reflect or suggest any relation to LD_50_, but to find a venom dose that was reliable, amenable to treatment and consistent with humane endpoints.

No LD_50_ estimate was made, and dose-finding was not intended to determine the relative potency of the venoms, but to serve as fit-for-purpose proof of concept based on the need to determine the efficacy of the study drugs (varespladib and antivenom) based on a dose of venom that reliably caused lethality (LD_100_). Dedicated studies of each venom type were conducted several months apart. To test whether varespladib or varespladib-methyl could rescue/mitigate envenoming effects, we first needed to determine doses of *Oxyuranus scutellatus* Australian coastal taipan and Papuan taipan venoms which were reliably lethal but consistently left sufficient survival time to achieve rescue or reverse weakness. Australian and Papuan venoms (0.03–1.0 mg/kg) were subcutaneously administered to juvenile pigs and the pigs were monitored for survival and clinical score as previously described (see Methods, Table 1) [36,43]. Animals were humanely euthanized with intravenous 39% sodium pentobarbital if they reached a clinical score of 5 for two consecutive evaluations or if they reached a clinical score of 6 at any one evaluation. This range of doses caused death and/or clinical scores causing euthanasia in all animals (Figure 1A,B). No animals required any additional analgesia after the mandatory first dose. For further experiments, a dose of 0.03 mg/kg was used for Australian taipan venom (mean survival time 331 min ± 15 min (Figure 1A) and 0.15 mg/kg for Papuan taipan venom (mean survival time 178 ± 31 min (Figure 1B). 

### 2.2. Rescue from Lethal Envenoming: Survival and Clinical Recovery from Weakness 

In a series of proof-of-concept experiments, we tested the abilities of oral varespladib-methyl or intravenous (IV) varespladib administered immediately (<5 min), 15 min, or 45 min after subcutaneous venom administration to prevent death and reverse neurotoxicity as compared to control animals. As described above, the doses of venom were chosen to test the efficacy of the intervention in a more clinically realistic model of envenoming than typically used for preclinical testing of antivenom efficacy [44]. Animals were given rescue treatment if they reached a clinical score of 5 (Table 1) for two consecutive evaluations or if they reached a clinical score of 6 at any one evaluation. If they did not respond to rescue treatment, they were humanely euthanized.

**Figure 2:**
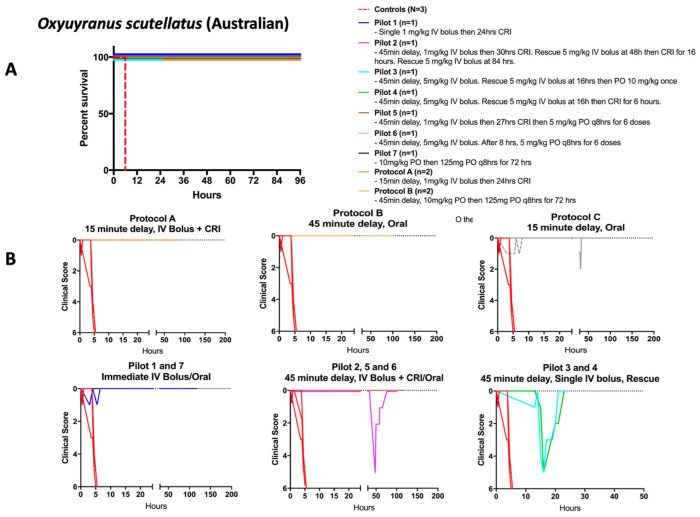
**Effect of varespladib on survival and weakness after Australian taipan venom**. (**A**) IV and oral varespladib administration resulted in sustained survival. (**B**) Clinical scores of 0 indicate no disability whereas 6 represents profound disability. When acute weakness or relapse occurred during planned pauses in drug administration, rapid rescue from that weakness was observed. In each instance, the nadir point is the time at which rescue doses of IV or oral varespladib were administered. Some animals were euthanized early (Pilot 3 and 4) for the purpose of examination of neuronal damage and interim tissue damage following resuscitation.

Thirteen pigs received Australian taipan venom and treatment with either IV or oral varespladib or both (Table 2, Figure 2). All animals receiving oral varespladib-methyl or IV varespladib were rescued from lethal doses of Australian taipan venom. Both forms of administration were equally effective at preventing death and reversing acute or recurrent neurotoxic signs (Figure 2). In those that did not receive any treatment, rapid deterioration ending in death within 1 and 5 h was the uniform outcome (*n* = 6, Figure 1). The dosage and timing of varespladib administration were varied to mimic various possible courses of treatment in clinical settings. We varied the dosage (1, 5, or 10 mg/kg), timing (immediate, 15-min delay, or 45-min delay), and length of continuous rate infusion (CRI) of varespladib (Table 2). Pigs treated immediately with either the oral or IV drug did not develop any sign of clinical deterioration based on the clinical score and survived the entire study period (*n* = 2, Pilot 1&7, Table 2). When varespladib administration was delayed by 15 or 45 min, relapses sometimes occurred; however, this weakness was readily reversed by restarting varespladib administration (Figure 2). Notably, varespladib by itself was sufficient to enable pigs to survive until the end of the study periods and both oral and IV administration of varespladib even more than four hours after envenoming were successful at preventing and reversing the neurotoxic or cardiovascular weakening effects of Australian taipan venom while the antivenom was unable to do so in this setting.

### 2.3. Varespladib and Antivenom 

**Figure 3:**
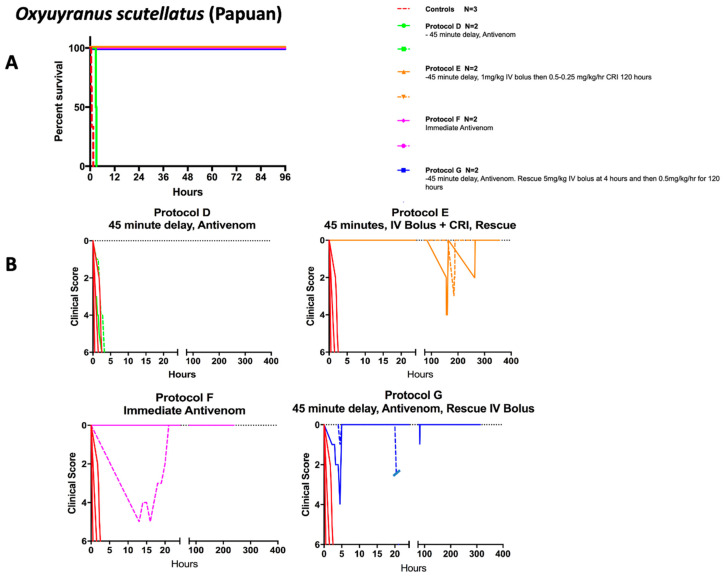
**Effect of varespladib and antivenom on survival and weakness after Papuan taipan venom.** (**A**) IV varespladib and immediate antivenom administration resulted in sustained survival. (**B**) Clinical scores of 0 indicate no disability whereas 6 represents profound disability. When acute weakness or relapse occurred during venom failure or planned pauses in drug administration, rapid rescue from that weakness was observed. In each instance, the nadir point is the time at which rescue doses of IV or oral varespladib dosage forms were administered. Some animals were euthanized early for the purpose of examination of neuronal damage and interim tissue damage following resuscitation. One animal had cardiovascular collapse at the time of antivenom administration and was resuscitated, receiving no further treatment (Protocol F), and one animal (Protocol G), was diagnosed with *Strep suis* and euthanized at 22 h after envenoming.

Following these proof-of-concept studies, we explored the efficacy of varespladib administration alone for Australian taipan Table 2 and Figure 2 and, as well as for Papuan taipan with, without, and in combination with antivenom treatment. As well, we examined whether varespladib could reverse weakness from neurotoxicity and/or hypotension following inadequate resuscitation with a single dose of antivenom (Table 3 and Figure 3). For these experiments, a monospecific taipan antivenom (CSL taipan specific) was administered by IV immediately (<5 min) or at a delay (45 min) following subcutaneous administration of Papuan taipan venom (0.15 mg/kg). Animals treated immediately with antivenom survived to the end of the study period (≥96 h, Protocol F, *n* = 2, Table 3), with one of the two pigs requiring CPR shortly after antivenom administration and having an indeterminate cause for its disability score that resolved spontaneously after approximately 24 h. For pigs treated with antivenom 45 min after venom was administered and *not* given varespladib, the mean survival time was approximately 3 h (178.5 min ± 31 min, Protocol D, Table 3 and Figure 3). Two animals receiving delayed antivenom administration were successfully rescued by varespladib (Protocol G, Table 3, and Figure 3); however, it was necessary to euthanize one of these animals because of identification of unexpected weakness, fever and, ultimately, culture-proven *Streptococcus suis* septicemia. The survival time for animals treated with varespladib was for the duration of the studies excepting the euthanized animal with septicemia (Protocols E, F, and G; Table 3 and Figure 3). No other animals showed signs or symptoms of septicemia. Three of five pigs receiving antivenom experienced anaphylaxis-like reactions not previously seen in previous experiments with mice or pigs using whole IgG antivenoms from a different manufacturer and lower protein concentration [35,36,45,46,47].

### 2.4. Pulmonary Findings in Animals Experimentally Envenomed with Papuan Taipan Venom

**Figure 4:**
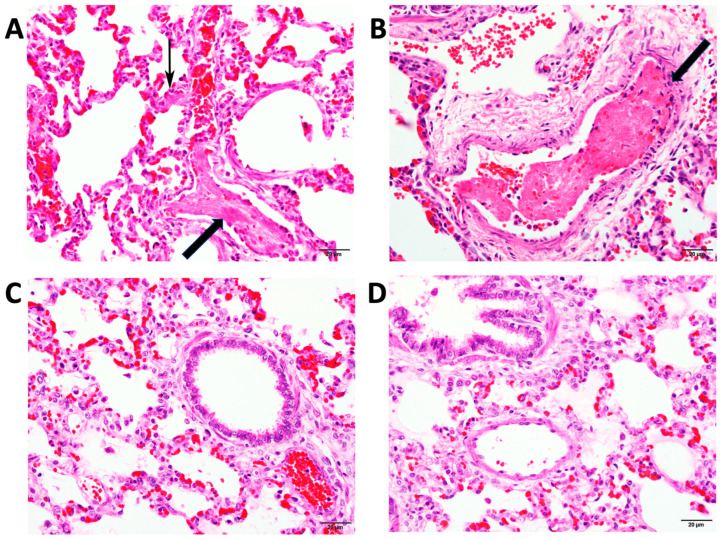
Representative images from histopathological examination of lung tissues at time of death. (**A**,**B**) Venom-only control animal with disseminated acute microthrombimicrothrombosesmicrothrombi and edema (large arrows) and septal capillaries occluded by fibrin thrombi (small arrow), (**C**) animal in protocol E treated with varespladib only and euthanized at 2 weeks, and (**D**) animal in Protocol G that did not respond to initial antivenom and received varespladib rescue also euthanized at 2 weeks. H&E stain. Magnification 400×. Useful comparison to histopathological examination of Papuan taipan venom in murine lung tissues can also be found in previous work by Herrera and colleagues [41].

Papuan taipan venom was of special interest as it contains non-sPLA2 pro-coagulant toxins [8,23,48,49,50]. Post-mortem histopathology was performed by a blinded, board-certified veterinary pathologist (JR) based on the experimental and clinical observation of pulmonary emboli observed in mice [41] and clinically in Papua New Guinea. Pulmonary emboli were present in 2/3 control animals and 2/2 animals receiving antivenom with a 45 min delay (Table 4). In comparison, animals receiving immediate treatment with antivenom (0/2) or treatment with varespladib (0/2) showed no pulmonary emboli (Table 4). Exemplary images provided by the blinded pathologist are shown in Figure 4. Tissue examination was not a feature of our previous study in mice experimentally envenomed with Papuan taipan venom [35]. 

## 3. Discussion

Our results from this early exploration of varespladib suggest that varespladib and varespladib-methyl could be efficacious components for the treatment of Australian coastal and Papuan taipan envenoming and should be considered for advancement to clinical trials in the Oceania region. Both oral and IV varespladib demonstrated an ability to reverse severe weakness and restore normal motor function when presented with these two taipan venoms. Overall, all but one animal treated with oral or IV drug survived. Importantly, we observed that administration of varespladib not only prevented the development of weakness when given early, but also prevented weakness and lethality when given at a delay significantly longer than antivenom was able to reverse. This was true both without antivenom and when weakness was resistant to antivenom, consistent with earlier studies in this pig model using *M. fulvius* venom [36]. These results are encouraging for exploring the utility of varespladib in the care of taipan envenoming, although, because these were pilot experiments with only a few animals for each group, further experiments would be necessary to fully understand each protocol’s advantages or disadvantages. 

Currently, the definitive treatment for snakebite envenoming requires acute care in health facilities and is dependent on the intravenous administration of animal-derived antivenoms. In this and previous studies, we observed that delayed antivenom treatment (given after 45 min in the pig model and one hour after envenoming in our mouse model) was ineffective. This agrees with clinical reports of less effective therapeutic performance by antivenoms in patients that have already developed signs of neurotoxicity prior to antivenom administration [51,52]. In contrast, immediate antivenom treatment prevented the development of weakness and this correlates with clinical observations indicating that patients receiving antivenom within 4 h are significantly less likely to develop life-threatening oropharyngeal neuromuscular paralysis [51,52]. By contrast, >60% of patients in whom antivenom administration is delayed more than 4 h require endotracheal intubation and mechanical ventilation—often for prolonged periods—to sustain life [51,52]. Not surprisingly, a large proportion of deaths from snakebites occur before access to or the decision to seek hospital care [1,53,54]. This is further exacerbated by structural factors such as poverty and distance to hospital care for most victims of snakebites [1,54,55,56]. These challenges underscore one of the limitations of antivenom therapy, especially in rural settings where the arrival to medical facilities may take several hours. Further, it highlights the importance of developing inexpensive, ideally orally administered therapies that could be used safely in the pre-hospital setting immediately following snakebite, with or without specific species identification or any special training. A challenge for antivenom therapies is the need for species-specific venoms in the absence of broadly neutralizing antibody formats available for clinical use. We have demonstrated preclinical efficacy of oral varespladib-methyl in a model of delayed treatment of lethal *Oxyuranus scutellatus* envenoming in mice [35], *Micrurus fulvius* (Eastern coral snake) envenoming in juvenile pigs [36] and now, for different geographic varieties of taipan venom juvenile pigs. Taken together, these results suggest that varespladib could be effective in improving outcomes against *Oxyuranus scutellatus* throughout its geographic range, but no human or veterinary clinical studies have yet been undertaken to investigate the role of varespladib in Australia/Oceania to date [57,58]. 

The domestic porcine model may be considered a stringent model to study gastrointestinal transit and absorption of drugs because *Sus domesticus* have amongst the slowest gastric emptying times of any mammal and may have provided a more robust challenge to the oral drug, varespladib-methyl, than using minipigs bred for their accelerated gastrointestinal clearance [59]. In all animals where drug dosing was intermittent, weakness waxed and waned, consistent with the reported half-lives of the drugs. Finally, we used doses of venom likely to be lower than what is normally encountered in a human taipan bite [8,40]. The pigs, even intubated, were exquisitely sensitive to the venoms such that this appears to be a limitation of the model for this venom, and the technique for injecting venom changed late in the study to avoid accidental intramuscular injection as appeared to have occurred on at least one occasion. Small differences in injection depth could theoretically affect the speed of onset (e.g., SQ vs. IM injection), but all pigs in the Papuan taipan study, unless treated immediately, showed signs of systemic toxicity during the study [51]. The long duration of the venom effect may have resulted from the venom’s intrinsic half-life or from being sequestered inside neuronal tissues or the fatty subcutaneous tissues of the pig allowing venom to leach, where it out for days and therefore require additional treatment [60]. Though not formally assessed, doses resulting in lethality appeared to be comparable to those for mice [35] suggesting that a murine model can be usefully applied as a translational model between species for this venom, given that the venom sample used was from the exact same individual snakes used in earlier mouse studies [35]. Nevertheless, not having an established lethality for the animal model *a priori* is a weakness of the study—limitations on budget, import of antivenom and Papuan taipan venom at the time of the studies notwithstanding. It is clear, in hindsight, it would have been appropriate to carry out this study with a repetition of the same experiments for each of the two venoms used. Not having established directly comparable lethality protocols early in the study may be considered a weakness. An additional limitation of this study was the inability to do formal neurophysiological testing given that arterial pressure monitors were not stable in free-roaming pigs. Only clinical examination by veterinarians was used to assess if the weakness was attributed by us to neurotoxicity, but unmeasured hypotension could have contributed to weakness [22,24,29]. In both preclinical and clinical studies, there is a need to better discriminate between neurotoxicity and cardiovascular instability [15,61,62]. We did not specifically examine mechanisms behind the reversal of weakness in this study, though this has recently been studied by Oliveira and colleagues in a mouse model [37]. Furthermore, several experiments were conducted with only one animal. In Protocol 3 and 4, for example, we were seeking scanning electron microscope (SEM) images of neuronal dendritic damage to compare to previous findings such as those reported by Prasarnpun [3,4], but did not succeed. Though unsuccessful with SEM, we think the clinical findings are worthy of reporting in this study and do not markedly diminish the conclusions. The observation that varespladib and antivenom might prevent death by neurotoxicity or pulmonary embolism in the Papuan taipan venom study neither suggests nor refutes a possible interaction between venom or endogenous sPLA2 with procoagulant venom SVMPs and/or SVSPs [41,50,63] because these were collected at the time of death and not at the same time point. Emboli might have been prevented or resolved in the interval. Nevertheless, the finding has potential and could possibly be developed to provide more insight into toxicities. Recent mechanistic studies by Salvador and Barbosa suggest a dual mechanism of active site inhibition and blockade of membrane docking [64,65] but these studies did not examine complex interactions between families of venom toxins such as those recently reviewed by Bickler [66].

These early studies suggest the potential of new therapeutics and highlight the need for better quantification of clinically relevant and translatable endpoints beyond mortality in both small and large animal models. Nevertheless, these results support continued investigation of the therapeutic potential of varespladib at both preclinical and clinical levels. Importantly, for both antivenom and varespladib, the observable benefit was seen by early treatment, further emphasizing the need to make treatments as accessible as possible, as soon as possible, to the victims of snakebite envenoming. Future studies with more systematic approaches for better comparison of venoms and their subtypes are needed. Factors that can improve insights in future studies of these venoms in animal models should include consistent times for tissue collections and, ideally, neurophysiological monitoring to better discriminate between weakness due to cardiovascular causes and primary neurotoxicity. Despite these limitations, this series of experiments highlights the potential for sPLA2 inhibition in the context of the complex envenoming syndromes by *Oxyuranus scutellatus,* and ongoing clinical studies may further enlighten the paths for future preclinical and clinical studies alike [15,57,58]. 

## 4. Materials and Methods

### 4.1. Venom, sPLA_2_ Inhibitor and Antivenom

The venom of adult specimens of *Oxyuranus scutellatus* was kindly provided by Dr. David Williams. Upon extraction, the venom was snap frozen and then freeze-dried. Venom solutions were prepared immediately before use by dissolving dry venom in 0.12 M NaCl, 0.04 M phosphate, pH 7.2 (PBS). Subcutaneous LD_50_ for this specific batch of venom was previously established by Herrera et al. [25,41] and is the same batch used in our previously published mouse study [35].

The sPLA_2_ inhibitors varespladib-methyl and sodium salt of varespladib were supplied by Ophirex, Inc. and prepared to 99.99% purity by ChemieTek (Indianapolis, Indiana). Varespladib-methyl was dissolved in 8% *w/v* gum arabic in water and the salt of varespladib was dissolved in water for injection at 10 mg/mL. The monospecific taipan antivenom produced by BioCSL (Australia) (Lot 07201; Expiry 11/2020; experiments concluded in February 2019) was used and shipped in a temperature-controlled manner to the study site with confirmation of even temperature throughout transit. It is a F(ab’)2 preparation obtained from the of plasma of horses immunized with the venom of *O. scutellatus* from Australia and preserved with 2 mg/mL phenol. Antivenom dosage was determined by administering 5 mL CSL antivenom which is equivalent to 1250 units of antivenom from a single 12,000-unit 47.6 mL vial. 1 unit of CSL antivenom per 0.01 mg venom. Animals weighed <10 kg and efficacy and sufficient dosage was further confirmed by administering antivenom immediately after known 100% lethal taipan venom dosage. The rate and route of administration were by the manufacturer’s recommendation. 

### 4.2. Animal Methodology

#### 4.2.1. Animals

A total of 32 female mixed-breed juvenile *Sus domesticus* were utilized in this study (23 for the Australian taipan and 9 for the examination of Papuan taipan venom). Pigs were acclimated to their new environment for 4–7 days prior to beginning the study. During this period all animals were visually examined by trained laboratory personnel twice daily for any signs of illness. None of the pigs exhibited signs of illness prior to beginning the project. All pigs were purchased through USDA-approved dealers. All animals were identified by ear tags and if tags were difficult to read, numbers were written on the pig’s side. Pigs were weighed on the morning of the study and given a complete physical examination by one of the study veterinarians. The mean weight in the *O. scutellatus* (Australian) control pigs was not different from treated pigs (control 20.47 ± 2.58 kg, treated 23.89 ± 3.92 kg (*p*-value = 0.18)). Study pigs in the Papuan taipan group were much smaller. The mean weight in control pigs was not different from treated pigs (control 9.17 ± 0.68 kg, treated 8.55 ± 0.73 kg (*p*-value = 0.23)). One pig was removed from the study after it became acutely septic with *Streptococcus suis* and was euthanized. No other animals exhibited signs or symptoms of illness. Two animals were sacrificed mid-experiment to understand the immediate consequences of drug withdrawal and to inspect neuromuscular junctions by electron microscopy, but this imaging effort was not successful.

#### 4.2.2. Housing

Swine were housed in a temperature-controlled environment in pens (14 square feet) with raised plastic flooring to minimize contact with urine and feces. A 12:12 light-dark cycle was maintained, and they were given ad libitum water and feed except for a 4–8 h fast prior to anesthesia induction. The pens allowed for individual or group housing depending on the phase of the study. During the acclimation period pigs were group housed. Once the intravenous catheters were placed, the pigs were individually housed to avoid the destruction of the catheters. Daily care of the pigs was provided by the centralized animal care unit, Animal Resources, as part of the Center for Veterinary Health Sciences’ animal care program accredited by AAALAC International. The study was conducted at Oklahoma State University, Veterinary Clinical Sciences, Stillwater, Oklahoma, United States.

#### 4.2.3. Anesthesia, Instrumentation, and Monitoring

Pigs were pre-medicated with midazolam (0.25 mg/kg) given intramuscularly in the semimembranosus/semitendinosus region using a 20-gauge 1-inch needle. Pigs were allowed to rest quietly in their pen for 15 min and become sedated. Once sedated, pigs were held in sternal recumbency, and anesthetized utilizing inhalant isoflurane gas administered through an anesthetic mask. Once anesthetized, an endotracheal tube was placed, and pigs were placed in dorsal recumbency for the catheterization procedure. Pigs were maintained on isoflurane throughout the procedure. Pigs had eye lubricant applied to each eye and were instrumented with an esophageal temperature probe and continuous electrocardiogram. Heart rate, respiratory rate, eye positioning, and temperature were monitored every 5 min to measure anesthetic depth. Once pigs were at an acceptable plane of anesthesia, intravenous catheters were placed into the jugular vein according to the following protocol. After the catheters were placed, pigs were placed in left lateral recumbency for easy access to the right forelimb where venom was injected. Once venom was injected treatment protocols were followed as stated below. 

#### 4.2.4. Intravenous Catheter Placement

With pigs in dorsal recumbency, the front legs were retracted caudally until they were close to parallel with the table and secured to the surgery table. Pigs were then clipped using #40 clipper blades and the area over the jugular veins was aseptically prepared. Ultrasound guidance was then used to place the intravenous j-wire catheter. Briefly, an 18–5 mHz linear probe was placed inside a sterile glove to enable the use of ultrasound while maintaining sterility, and the jugular vein was visualized. A 5 fr 13 cm double lumen central line catheter made by Arrow, was inserted using the standard Seldinger technique with the guide needle for the over-the-wire catheter placed under ultrasound guidance. Electrocardiogram was monitored during catheter placement for any evidence of arrhythmias. If arrhythmias were seen, the catheter was backed out of the vein until it disappeared. Catheters were secured in place utilizing 0 PDS suture and the catheters were wrapped with 2-inch Elastikon to further secure them and prevent inadvertent removal. 

#### 4.2.5. Analgesia

Buprenorphine (0.01 mg/kg) was administered intramuscularly (IM) in the cervical musculature once pigs were intubated and prior to placing the intravenous catheters. All pigs then received an additional dose of buprenorphine at 4 h post venom. Pigs were evaluated for pain by a lameness scoring system and, if necessary, were continued on buprenorphine every 4 h until it was no longer needed. Lameness score of 3 (Table 5) or higher warranted analgesic administration. 

#### 4.2.6. Euthanasia

Pigs were euthanized or given rescue treatment if they reached a clinical score of 5 (Table 1) for 2 consecutive evaluations or if they reached a clinical score of 6 at any one evaluation and did not respond to rescue treatment. They were humanely euthanized using 39% sodium pentobarbital given intravenously. All pigs were submitted for postmortem evaluation.

### 4.3. Envenoming

Venom was injected using a 26-gauge, 0.5-inch (12.7 mm) needle connected to a luer lock 3 mL syringe, in the distal lateral portion of the right antebrachium of the subject animals. The needle was inserted approximately 3 mm below the skin into the subcutaneous tissues and venom was injected. The total volume injected did not exceed 1 mL (range 0.4–0.9 mL). The same veterinarian (LLG) administered every dose of venom throughout the study period. 

### 4.4. Lethality Dose Finding

Dose finding was performed to find a lethal venom dose and only intended as fit for purpose and not to establish an LD_50_. The time course to meet euthanasia criteria was followed to select a dose of venom for the remainder of the study. The desired dose was intended to be fatal within 3 to 8 h of experimental envenoming. Starting doses were based on previous studies as well as the venom yields of adult snakes. For both subspecies, the starting dose was 0.3 mg/kg. The final dose selected for Australian *O. scutellatus* was 0.03 mg/kg and 0.15 mg/kg for *O. scutellatus* from PNG although this dose turned out to be more rapidly fatal than planned. 

### 4.5. Study Period Detail

Pigs were monitored throughout the study period for clinical signs of pain (lameness score (Table 1 and Table 5) modified from the Obel laminitis grading system for horses and the AAEP lameness scale) [36,43] and neurotoxicity [36]. Evaluations were made according to the following schedule:

- Every 15 min post-venom administration for the first 4 h following recovery from general anesthesia.
- Every 30 min from 4–8 h post-venom administration.
- Hourly from 8 h to 48 h post-venom administration.
- Every 6 h from 48 h through 96 h or study end.

All monitoring was done in person until 48 h. After 48 h, if pigs were asymptomatic and were not on a constant rate infusion, they were observed in person twice daily and monitored by video for the other time points. If pigs were noted to be showing signs of pain (lameness score of 3 or greater) or neurotoxicity (clinical score of 4 or greater), one of the study veterinarians examined the pig in person within 1 h of noting abnormal signs. 

### 4.6. Rescue Experiments with Varespladib and Antivenom

Several rescue protocols, described in Table 3 and Table 4, were used after envenoming. Briefly, the ability of the sPLA_2_ inhibitors to prevent or treat venom-induced coagulopathy and neurotoxicity was assessed in proof-of-concept efficacy experiments and in scenarios designed to potentially model clinical use alone. IV drugs were administered either as 10–15 min rapid infusions “boluses”, continuous rate infusion (CRI), or a combination. Oral drugs were administered via orogastric tube as needed, oral “treat”, or a combination during the experiments.

### 4.7. Tissue Pathology

Tissues were collected immediately postmortem and fixed in formalin and stained with hematoxylin and eosin for examination by a board-certified veterinary pathologist blind to the treatment groups. 

## Figures and Tables

**Table 1:** Clinical score used in the assessment of envenoming severity [36,43].

Score	Clinical Condition
0	Normal activity, interested in food/water/toys, normal grunting, curious about environment, responds normally to stimulation by moving away, rises easily and quickly from recumbency when stimulated.
1	Normal movement around pen, reduced interest in food/water/toys, reduced interaction with environment or caretakers, rises from recumbency and moves away when stimulated.
2	Evidence of weakness in one or more limbs, reduced interest in food/water/toys, reduced responsiveness to stimulation but still able to rise normally from recumbency.
3	Significant evidence of weakness (dog sitting) but able to rise unassisted when stimulated, some interest in food/water/toys, can stay up for >3 min.
4	Significant evidence of weakness (dog sitting), requires assistance to rise but can still stand, stays up >15 s but less than 3 min.
5	Significant evidence of weakness (dog sitting), requires assistance to stand but unable to remain standing for >15 s.
6	Marked evidence of weakness (sternal or lateral recumbency) unable to rise even with assistance.

**Table 2:** Varespladib dosage and timing in Australian coastal taipan (no antivenom).

Protocol (# of Animals)	Treatment	Initial Dosage Administration	Result
Pilot 1 (*n* = 1)	Venom + IV varespladib	-Immediate loading dose -1 mg/kg over 15 min -CRI for 20 h	-Survival to end of study period
Pilot 2 (*n* = 1)	Venom + IV varespladib	-45 min delay -loading dose 1 mg/kg over 15 min -CRI for 30 h	-Relapse at 36 h, rescue IV bolus 5 mg/kg -Relapse at 48 h, rescue IV bolus 5 mg/kg followed by 16 h CRI -Survival to end of study period
Pilot 3 (*n* = 1)	Venom + IV varespladib with switch to oral	-45 min delay, -loading dose 5 mg/kg over 15 min	-Relapse at 16 h, rescue IV bolus 5 mg/kg and Oral 10 mg/kg × 1 -Survival to end of study period
Pilot 4 (*n* = 1)	Venom + IV varespladib	-45 min delay -loading dose 5 mg/kg over 15 min	-Relapse at 16 h, rescue IV bolus 5 mg/kg followed by 6 h CRI -Survival to end of study period
Pilot 5 (*n* = 1)	Venom + IV varespladib and switch to oral varespladib	-45 min delay, loading dose 1 mg/kg over 15 min -CRI for ~27 h -Oral 5 mg/kg every 8 h for 48 h	-Survival to end of study period
Pilot 6 (*n* = 1)	Venom + IV varespladib and switch to oral varespladib	-45 min delay, loading dose 5 mg/kg over 15 min -Wait 8 h -Oral 5 mg/kg every 8 h	-Relapse at 105 h, rescue IV bolus 5 mg/kg and oral 5 mg/kg q8 h x6 -Survival to end of study period
Pilot 7 (*n* = 1)	Venom + Oral varespladib	-Immediate orogastric (OG) tube 10 mg/kg -Wait 8 h -Oral 5 mg/kg every 8 h for 72 h	-Survival to end of study period
Protocol A (*n* = 2)	Venom + IV varespladib	-15 min delay, loading dose 1 mg/kg over 15 min -CRI for 23 h and 25 h	-Survival to end of study period
Protocol B (*n* = 2)	Venom + Oral varespladib	-45 min delay, OG tube 10 mg/kg -Wait 8 h -Oral 5 mg/kg every 8 h for 72 h	-Survival to end of study period
Protocol C (*n* = 2)	Venom + Oral varespladib	-15 min delay, OG tube 5 mg/kg -Wait 8 h -Oral 5 mg/kg every 8 h for 72 h	-Survival to end of study period

**Table 3:** Varespladib (IV) and antivenom administration in Papuan taipan.

Protocol (# of Animals)	Treatment	Initial Dosage Administration	Results
Protocol D (*n* = 2)	Venom + antivenom	-45 min delay -Antivenom 5 mL diluted in 25 mL LRS given over 10 min per manufacturer’s instructions	Euthanasia at 2.8 and 3.1 h, respectively
Protocol E (*n* = 2)	Venom + IV varespladib	-45 min delay, loading dose 1 mg/kg IV over 15 min -CRI (0.25–0.5 mg/kg/h) for 140–167 h	-Relapse 17–18 h later, rescue with IV bolus 1 mg/kg over 10 min -Survival to end of study
Protocol F (*n* = 2)	Venom + antivenom	-Immediate antivenom 5 mL diluted in 25 mL LRS given over 15 min	-Survival to end of study
Protocol G (*n* = 2)	Venom + antivenom + IV varespladib (rescue)	-45 min delay, antivenom 5 mL diluted in 25 mL LRS given over 28 min -CRI until 145 h post venom with rescue IV bolus *prn* weakness	Surviving animal: Severe signs/symptoms at 4.2 h post venom and 3.5 h after antivenom, rescue with single IV rescue bolus 5 mg/kg at 81 h and continuation of CRI. Survival to end of study. Euthanized animal: Milder signs/symptoms at approximately 4 h. Euthanized at 22 h for confirmed *S. suis* infection

**Table 4:** Presence of pulmonary emboli upon post-mortem examination.

Treatment Group	Protocol	Animals with Identified Pulmonary Emboli
Venom only (Control)	Control	2 of 3
Venom + antivenom (45 min delay)	D	2 of 2
Venom + varespladib (45 min delay)	E	0 of 2
Venom + antivenom (immediate)	F	0 of 2
Venom + antivenom (45 min delay) + varespladib rescue	G	1 of 2

**Table 5:** Clinical lameness score is used to assess the need for repeated analgesia [36,43].

Lameness Score	Clinical Picture
0	No abnormalities noted in ability to stand or walk
1	Slight limp or favoring of limb(s) noted while walking but stands normally
2	Slight limp or favoring of limb(s) noted while walking and favoring of limb(s) apparent when standing
3	Significant limp or favoring of limb (s) when walking, bears minimal weight on limb(s) when standing
4	Reluctant, but able/willing to bear weight when standing or walking
5	Unable/unwilling to bear weight on limb (s)
